# Reversed halo sign in pneumocystis pneumonia: a case report

**DOI:** 10.1186/1471-2342-10-26

**Published:** 2010-11-23

**Authors:** Hiroshi Otera, Kimihide Tada, Toshiyasu Sakurai, Kimio Hashimoto, Akihiko Ikeda

**Affiliations:** 1 Department of Respiratory Medicine, Nishi-Kobe Medical Center 5-7-1, Kojidai, Nishi-ku, Kobe, 651-2273 Japan; 2 Department of Pathology, Nishi-Kobe Medical Center 5-7-1, Kojidai, Nishi-ku, Kobe, 651-2273 Japan

## Abstract

**Background:**

The reversed halo sign may sometimes be seen in patients with cryptogenic organizing pneumonia, but is rarely associated with other diseases.

**Case presentation:**

We present a case study of a 32-year-old male patient with acquired immunodeficiency syndrome, who had previously been treated with chemotherapy for non-Hodgkin's lymphoma. A chest X-ray showed bilateral patchy infiltrates. High-resolution computed tomography revealed the reversed halo sign in both upper lobes. The patient was diagnosed with pneumocystis pneumonia, which was successfully treated with sulfamethoxazole trimethoprim; the reversed halo sign disappeared, leaving cystic lesions. Cases such as this one are rare, but show that the reversed halo sign may occur in patients who do not have cryptogenic organizing pneumonia.

**Conclusion:**

Physicians can avoid making an incorrect diagnosis and prescribing the wrong treatment by carefully evaluating all clinical criteria rather than assuming that the reversed halo sign only occurs with cryptogenic organizing pneumonia.

## Background

Pneumocystis pneumonia (PCP) is the most common serious respiratory complication found in patients with acquired immunodeficiency syndrome (AIDS) [1]. The typical radiographic features of PCP are bilateral perihilar distributions of ground-glass opacities (GGOs); however, a wide variety of radiographic findings have been observed [2-4]. The reversed halo sign is reported to be specifically associated with cryptogenic organizing pneumonia (COP) [5], but here we present the first case, to our knowledge, of PCP with a reversed halo sign.

## Case presentation

**Figure 1:**
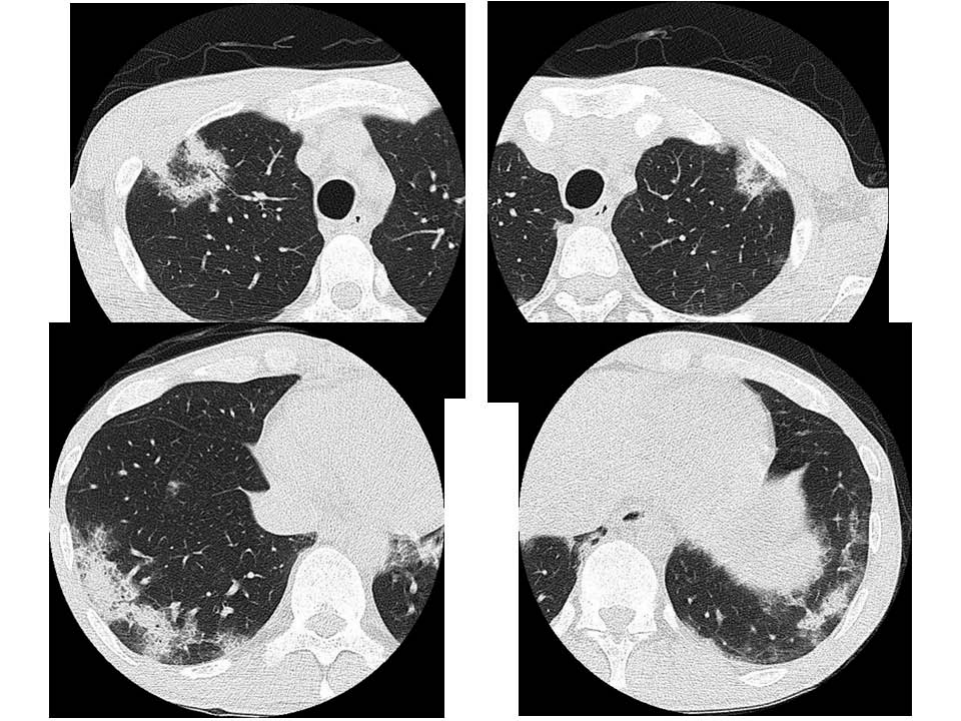
**High-resolution computed tomography images of the lung before sulfamethoxazole trimethoprim treatment.** The reversed halo signs and consolidations are shown.

**Figure 2:**
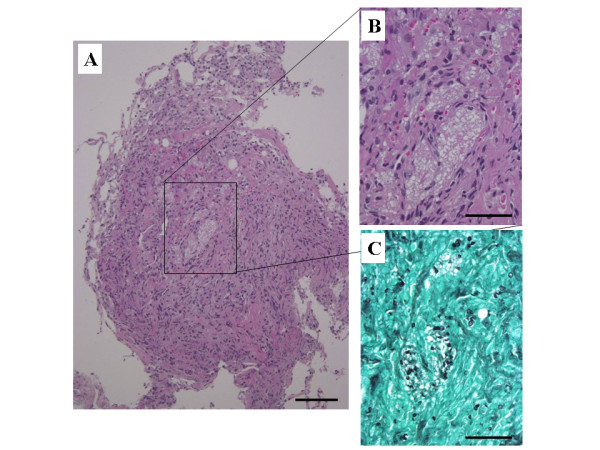
**Lung specimen obtained by transbronchial lung biopsy**. Pathological findings of the specimen show many cysts and intra-alveolar exudates. (A, internal scale bar = 100 μm hematoxylin and eosin stain. B, internal scale bar = 50 μm hematoxylin and eosin stain. C, internal scale bar = 50 μm Grocott stain.)

A 32-year-old man was admitted to our hospital with an eight-week history of fever and productive cough. Approximately two years prior to presentation, extra-nodal B-cell non-Hodgkin's lymphoma (NHL) had been diagnosed, for which the patient had undergone chemotherapy. On admission, the patient's body temperature was 38.8°C, blood pressure was 124/72 mm Hg, pulse rate was 106 beats/min, and percutaneous oxygen saturation was 96% in room air. Physical examination revealed coarse crackles over the posterior right lung. The patient's white blood count was 5,500 cells/mm^3 ^(lymphocytes = 25%, neutrophils = 55%, and atypical = 8%). CD4 cell count was 53 cells/μl and HIV-1 RNA count was 2.5 × 10^4 ^copies/ml. Chest radiography showed bilateral patchy infiltrates. A high-resolution computed tomography (HRCT) scan showed the reversed halo sign in the upper lobes and patchy parenchymal infiltrates in the lower lobes (Figure 1). A transbronchial lung biopsy was performed from the upper segmental bronchus. Histologically, the Grocott stain showed characteristic staining of many round structures surrounded by foamy, intra-alveolar exudate and unaccompanied by interstitial pneumonitis (Figure 2).

**Figure 3:**
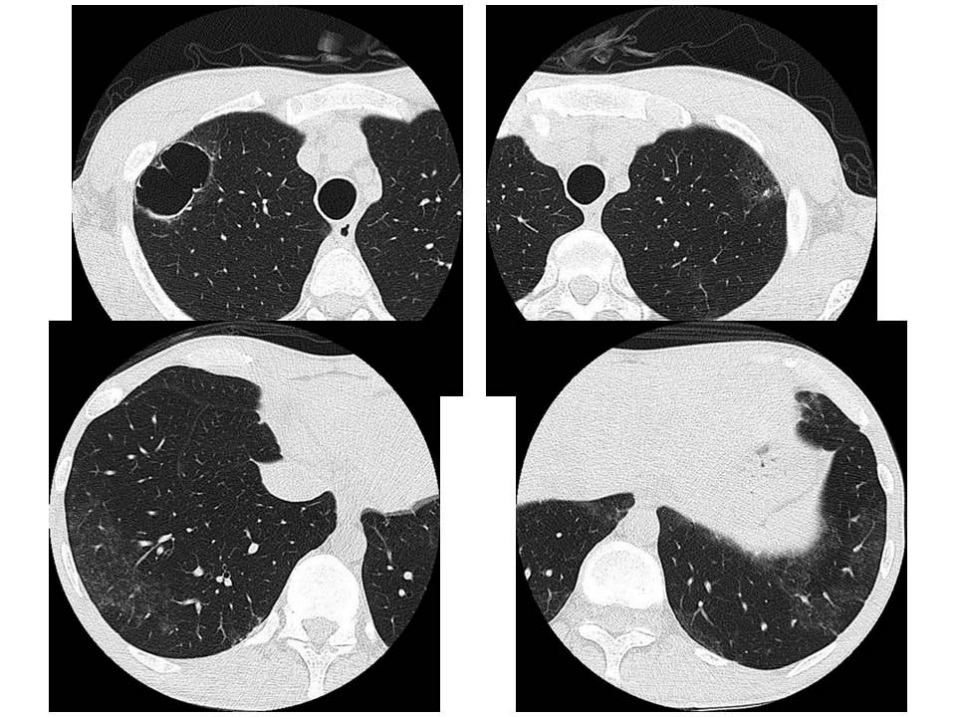
**High-resolution computed tomography images of the lung 20 days after sulfamethoxazole trimethoprim treatment**. Various sizes of cystic lesions are seen in the bilateral upper lobes and small nodules are seen in the bilateral lower lobes.

The patient was diagnosed with PCP and AIDS. He was treated with sulfamethoxazole trimethoprim. The symptoms improved within 20 days, and chest radiography showed normal and chest CT showed remarkable improvements; the reversed halo sign disappeared, leaving cystic lesions (Figure 3).

## Discussion

In 1996, Voloudaki et al. [6] reported two cases of COP that appeared on HRCT scans as central GGO surrounded by a denser air-space consolidation of crescent and ring shapes. This appearance was later named the "reversed halo sign" and defined as central GGO surrounded by a denser consolidation of crescentic (forming more than three-fourths of a circle) or ring (forming a complete circle) shapes that are at least 2 mm in thickness [5]. In studies of patients with COP, the reverse halo sign has been found in 12-19% of patients [5,7] (Table 1). In fact, the reversed halo sign is so closely associated with COP that its presence is commonly used to make a diagnosis.

**Table 1:** Summary of reported cases of Reversed Halo Sign

Diagnosis	Number of patients (%)	[Ref.]
Bronchiolitis obliterans organizing pneumonia	2	[6]
Sarcoidosis	1	[8]
Cryptogenic organizing pneumonia	6/31 (19%)	[5]
Paracoccidioidomycosis	15/148 (10%)	[10]
Cryptogenic organizing pneumonia	2/34 (5.9%)	[7]
Secondary organizing pneumonia (of 1 breast cancer and 1 rheumatoid arthritis patient)	2/34 (5.9%)	[7]
Paracoccidioidomycosis	15/77 (19%)	[13]
Lymphomatoid granulomatosis	1	[14]
Tuberculosis	1	[15]
Lipoid pneumonia	1	[16]
Wegener's granulomatosis	1	[17]
Zygomycosis	7/37 (19%)	[9]
Invasive pulmonary aspergillosis	1/132 (<0.7%)	[9]
Sarcoidosis	1	[18]
Sarcoidosis	1	[19]
Tuberculosis	1	[20]
Cryptogenic organizing pneumonia	1	[21]
Pneumocystis pneumonia	1	present

In Voloudaki's study of COP [6], the central GGO corresponded histopathologically to the area of alveolar septal inflammation and cellular debris; the ring-shaped or crescentic peripheral air-space consolidation corresponded to the area of organizing pneumonia within the alveolar ducts. The mechanism behind the reversed halo sign, in association with non-infectious diseases, has been likened to dropping a pebble into a pond: Inflammation occurs in the middle and spreads outward, while simultaneously improving at the site of occurrence. The changes in the center of the "pond" result in the GGO appearance [8].

The reversed halo sign has also been observed in association with other diseases, including the infectious diseases paracoccidioidomycosis (PCM), tuberculosis (TB), pulmonary zygomycosis, and pulmonary aspergillosis. In fungal infections, histology revealed that the lung was infarcted and had more hemorrhaging in the periphery than in the center [9]. However, in patients with PCM who underwent surgical lung biopsy, the central area of the lesions consisted of an inflammatory infiltrate in the alveolar septa, composed of macrophages, lymphocytes, plasma cells, and some giant cells, with relative preservation of the alveolar spaces [10]. The peripheries of the lesions consisted of dense and homogeneous intra-alveolar cellular infiltrates. There was no evidence of organizing pneumonia or large hemorrhage. Grocott-Gomori methenamine-silver stain confirmed the presence of fungus (*P. brasiliensis*) in the alveolar septa and in the air spaces. In TB cases, pathology revealed the presence of caseating granulomas. The pathological findings for each infectious disease are distinct, greatly diminishing the chance of a misdiagnosis.

In humans, PCP is caused by infection with *Pneumocystis jiroveci*. This pathogen exists in two dominant forms: a cyst and a trophozoite. In humans, the trophozoite attaches itself to type 1 alveolar epithelial pneumocytes by way of interdigitation with the host cell membrane. This causes proteinaceous fluid to leak into the alveolar spaces. These spaces become filled with a characteristic "foamy" eosinophilic exudate that contains trophozoites, cysts, fibrin, and dead cells. Type 2 pneumocytes replicate in order to replace the damaged type 1 pneumocytes. This leads to mobilization of lung macrophages and plasma cells within the interstitium, causing a mild interstitial pneumonitis. Eventually, interstitial fibrosis develops as the lung tries to repair itself [2]. This process produces the GGO and consolidation findings that can be observed with HRCT.

In the case presented here, pathological findings showed an inflammatory infiltrate in the alveolar space, without a great amount of hemorrhage, and unassociated with organizing pneumonia. PCP and PCM have many pathological and biological similarities. Thus, the mechanism causing the reversed halo sign in the two diseases may be the same, possibly stemming from the host's immune response or the biological features of mycosis.

To our knowledge, PCP has never been reported in connection with the reversed halo sign. PCP occurs not only in patients with AIDS, but also in individuals with other malignant diseases; the radiological features of PCP differ depending on patients' exact medical histories [11]. For instance, patients with AIDS presented GGO without consolidation on CT [11]. However, patients with malignancy were likely to experience PCP with diffuse GGO with inhomogeneous distribution unrelated to secondary lobes, and consolidation with GGO [11]. Additionally, PCP with malignancy has been observed in conjunction with nodular infiltration [12]. Consolidation in HIV-negative patients may be associated with severe inflammation of the lung. In the case study presented here, the reversed halo sign with HIV-PCP is induced by the special condition of the host's immune system; his underlying diseases, including NHL and HIV, are likely to have influenced the development of the disease. The HIV infection is also likely to have been responsible for the cystic lesions that remained after the reversed halo sign disappeared, though this characteristic is much more commonly observed in PCP patients with AIDS [2].

## Conclusions

The reversed halo sign does not appear to be specific for COP, and may be seen in patients with any active infection. This is likely because there are a number of different mechanisms that can cause this characteristic pattern. In this case, the reversed halo sign is likely to have been found in association with PCP because of the patient's infection with HIV. Because the reversed halo sign can indicate different diseases that require very different treatments, physicians should not assume a diagnosis of COP whenever patients present with this trait.

## Consent

Written informed consent was obtained from the patient for publication of this case report and any accompanying imagings. A copy of the written consent is available for review by the Editor-in-chief of this journal.

## Competing interests

The authors declare that they have no competing interests.

## Authors' contributions

HO was in charge of the overall care of the patient, performed the literature search, compiled data and drafted the manuscript. KT, TS and AI provided intellectual input and revised the manuscript. KH has participated in the histological diagnosis of the case. All authors have read and approved the final manuscript.

## Authors' information

Departments of ^1^Respiratory Medicine and ^2^Pathology, Nishi-Kobe Medical Center

5-7-1, Kojidai, Nishi-ku, Kobe, 651-2273 Japan

## Pre-publication history

The pre-publication history for this paper can be accessed here:

http://www.biomedcentral.com/1471-2342/10/26/prepub

## References

[B1] Thomas CF Jr Limper AH Pneumocystis pneumonia New Engl J Med 2004 350 2487 2498 10.1056/NEJMra032588 15190141

[B2] Kuhlman JE Pneumocystic infections: The radiologist's perspective Radiology 1996 198 623 635 8628844 10.1148/radiology.198.3.8628844

[B3] Boiselle PM Crans CA Kaplan MA The changing face of Pneumocystis carinii Pneumonia in AIDS patients AJR 1999 172 1301 1309 10227507 10.2214/ajr.172.5.10227507

[B4] Fujii T Nakamura T Iwamoto A Pneumocystis pneumonia in patients with HIV infection: clinical manifestations, laboratory findings, and radiological features J Infect Chemother 2007 13 1 7 10.1007/s10156-006-0484-5 17334722

[B5] Kim SJ Lee KS Ryu YH Yoon YC Choe KO Kim TS Sung KJ Reversed halo sign on high-resolution CT of cryptogenic organizing pneumonia: diagnostic implications AJR 2003 180 1251 1254 12704033 10.2214/ajr.180.5.1801251

[B6] Voloudaki AE Bouros DA Froudarakis ME Datseris GE Apostolaki EG Gourtsoyiannis NC Crescentic and ring-shaped opacities. CT features in two cases of bronchiolitis obliterans organizing pneumonia (BOOP) Acta Radiol 1996 37 889 892 10.3109/02841859609175463 8995460

[B7] Soberón AB Sánchez MIT Rio FG Almaraz CS Pajares MP Rodoíquez MP High resolution computed tomography patterns of organizing pneumonia Arch Bronchoneumol 2006 42 413 416 10.1016/S1579-2129(06)60557-0 16948996

[B8] Marlow TJ Krapiva PI Schabel SI Judson MA The "fairy ring": a new radiographic findings in sarcoidosis Chest 1999 115 275 276 10.1378/chest.115.1.275 9925098

[B9] Wahba H Truong MT Lei X Kontoyiannis DP Marom EM Reversed Halo Sign in Invasive Pulmonary Fungal Infections Clin Infect Dis 2008 46 1733 1737 10.1086/587991 18419427

[B10] Gasparetto EL Escuissato DL Davaus T de Cerqueira Elza Maria FP Souza AS Jr Marchiori E Müller NL Reversed halo sign in pulmonary paracoccidioidomycosis AJR 2005 184 1932 1934 15908556 10.2214/ajr.184.6.01841932

[B11] Tasaka S Tokuda H Sakai F Fujii T Tateda K Johkoh T Ohmagari N Ohta H Araoka H Kikuchi Y Yasui M Inuzuka K Goto H Comparison of Clinical and Radiological Features of *Pneumocystis *Pneumonia Between Malignancy cases and Acquired Immunodeficiency Syndrome Cases: A Multicenter Study Inter Med 2010 49 273 281 10.2169/internalmedicine.49.2871 20154431

[B12] Torres HA Chemaly RF Storey R Aguilera EA Nogueras GM Safdar A Rolston KVI Raad II Kontoyiannis DP Influence of type of cancer and hematopoietic stem cell transplantation on clinical presentation of *Pneumocystis jiroveci *pneumonia in cancer patients Eur J Clin Microbiol Infect Dis 2006 25 382 388 10.1007/s10096-006-0149-4 16767486

[B13] Souza AS Gasparetto EL Davaus T Escuissato DL Marchiori E High-Resolution CT Findings of 77 Patients with Untreated Pulmonary Paracoccidioidomycosis AJR 2006 187 1248 1252 10.2214/AJR.05.1065 17056912

[B14] Benamore RE Weisbrod GL Hwang DM Bailey DJ Pierre AF Lazar NM Maimon N Reversed halo sign in lymphomatoid granulomatosis Br J Radiol 2007 80 956 e162 6 10.1259/bjr/46361210 17762047

[B15] Ahuja A Gothi D Joshi JM A 15-year-Old Boy with "Reversed Hallo" Indian J Chest Dis Allied Sci 2007 49 99 101

[B16] Kanaji N Bandoh S Nagamura N Chang SS Ishikawa S Yokomise H Katsuki N Haba R Kushida Y Ishida T Lipid pneumonia showing multiple pulmonary nodules and reversed halo sign Respir Med Extra 2007 3 98 101 10.1016/j.rmedx.2007.04.002

[B17] Agarwal R Aggarwal AN Gupta D Another cause of reversed halo sign: Wegener's granulomatosis Br J Radiol 2007 80 849 850 10.1259/bjr/61353689 17959924

[B18] Kumazoe H Matsunaga K Nagata N Komori M Wakamatsu K Kajiki A Nakazono T Kudo S "Reversed halo sign" of high-resolution computed tomography in pulmonary sarcoidosis J Thorac Imaging 2009 24 66 68 10.1097/RTI.0b013e318190476f 19242310

[B19] Marchiori E Zanetti G Mano CM Hochhegger B Irion KL The reversed halo sign: another atypical manifestation of sarcoidosis Korean J Radiol 2010 11 251 252 10.3348/kjr.2010.11.2.251 20191076 PMC2827792

[B20] Marchiori E Grando RD Simões Dos Santos CE Maffazzioli Santos Balzan L Zanetti G Mano CM Gutierrez RS Pulmonary tuberculosis associated with the reversed halo sign on high-resolution CT Br J Radiol 2010 83 e58 60 10.1259/bjr/22699201 20197429 PMC3473561

[B21] Maimon N A 47-year-old female with shortness of breath and "reversed halo sign" Eur Respir Rev 2010 19 83 85 10.1183/09059180.00005109 20956171 PMC9491645

